# Iodide of Potassium in Diseases of Brain in Children

**Published:** 1860-05

**Authors:** 


*Iodide of Potassium in Diseases of the Brain in Children.*—In the *Boston Medical and Surgical Journal* for January 19th, is an article upon the above subject, by John Coldstream, M.

D., &c. copied from the *Edinburg Medical and Surgical Journal.* Dr. Coldstream says that for more than twenty years he has used the iodide of potassium in brain disturbances of children particular in hydrocephalus. He says, "The results I have obtained have been so much more decidedly favorable than those which I had been accustomed to see under the employment of depletition, calomel, and purgatives, that I have been surprised to find comparatively few references to the treatment of diseases of the head by this agent in the more recent works on the practice of medicine. I have met with but a small number of practitioners who seem to recognize it as a remedy of marked efficacy." Following the above remarks, Dr. Coldstream makes allusion to, and quotes the opinions of those who have spoken well of this remedy in this class of diseases.

